# Bone cement reinforcement improves the therapeutic effects of screws in elderly patients with pelvic fragility factures

**DOI:** 10.1186/s13018-024-04666-3

**Published:** 2024-03-18

**Authors:** Lecai Gao, Baorui Xing

**Affiliations:** Department of Orthopaedic Surgery, Hebei Cangzhou Hospital of Integrated Traditional Chinese Medicine and Western Medicine, Cangzhou, Hebei 061000 China

**Keywords:** Bone cement, Pelvic fragility factures, Robotic technology

## Abstract

**Background:**

Pelvic fragility fractures in elderly individuals present significant challenges in orthopedic and geriatric medicine due to reduced bone density and increased frailty associated with aging.

**Methods:**

This study involved 150 elderly patients with pelvic fragility fractures. The patients were divided into two groups, the observation group (Observation) and the control group (Control), using a random number table. Artificial intelligence, specifically the Tianji Orthopedic Robot, was employed for surgical assistance. The observation group received bone cement reinforcement along with screw fixation using the robotic system, while the control group received conventional screw fixation alone. Follow-up data were collected for one-year post-treatment.

**Results:**

The observation group exhibited significantly lower clinical healing time of fractures and reduced bed rest time compared to the control group. Additionally, the observation group experienced less postoperative pain at 1 and 3 months, indicating the benefits of bone cement reinforcement. Moreover, patients in the observation group demonstrated significantly better functional recovery at 1-, 3-, and 6-months post-surgery compared to the control group.

**Conclusion:**

The combination of bone cement reinforcement and robotic technology resulted in accelerated fracture healing, reduced bed rest time, and improved postoperative pain relief and functional recovery.

## Introduction

Pelvic fragility fractures in the elderly present a significant clinical challenge in orthopedic and geriatric medicine [1, 2]. In fact, age is one of the most significant risk factors for fracture [3]. These fractures, commonly occurring due to reduced bone density and increased frailty associated with aging, can lead to substantial morbidity and mortality among older adults [4, 5]. Pelvic fragility fractures are typically caused by low-energy trauma, such as a fall from standing height, and are closely associated with osteoporosis and age-related decline in bone strength [6]. The management of pelvic fragility fractures in the elderly necessitates a patient-centered approach, considering individual medical conditions, comorbidities, and functional status [7, 8]. Non-surgical management, including pain control, early mobilization, and rehabilitation, may be appropriate for stable fractures in select patients [9]. However, unstable fractures often require surgical intervention, such as percutaneous fixation or open reduction with internal fixation (ORIF), to restore pelvic stability and promote early recovery [10, 11].

The bone cement technique, also known as cementation or cemented fixation, is a fundamental and widely used procedure in orthopedic surgery [12]. This technique involves the application of bone cement, commonly known as polymethylmethacrylate (PMMA), to facilitate the fixation of implants and prostheses within bone structures [13]. PMMA is a biocompatible, non-toxic, and stable material that exhibits high strength and excellent bonding capabilities with bone [14]. The availability of PMMA in various viscosities and antibiotic-impregnated formulations enhances its versatility for different clinical applications [15]. The bone cement technique represents a remarkable advancement in orthopedic surgery, significantly improving patient outcomes in joint arthroplasty, fracture management, and revision surgeries. The cemented fixation method has provided excellent stability and promoting successful long-term outcomes for patients, especially the elder [16].

Clinical studies evaluating the efficacy of bone cement in pelvic fragility fractures have shown promising results, with improved pain relief, early mobilization, and enhanced functional recovery observed in many cases [6, 17]. However, further research is warranted to establish standardized guidelines, identify optimal cement formulations, and explore long-term outcomes in this specific patient population. Therefore, the purpose of this study is to analyze the application effect of bone cement reinforcement technology assisted by artificial intelligence (Tianji Orthopedic Robot) in the treatment of elderly fragility pelvic fractures.

## Materials and methods

### Study design and participants

**Fig. 1:**
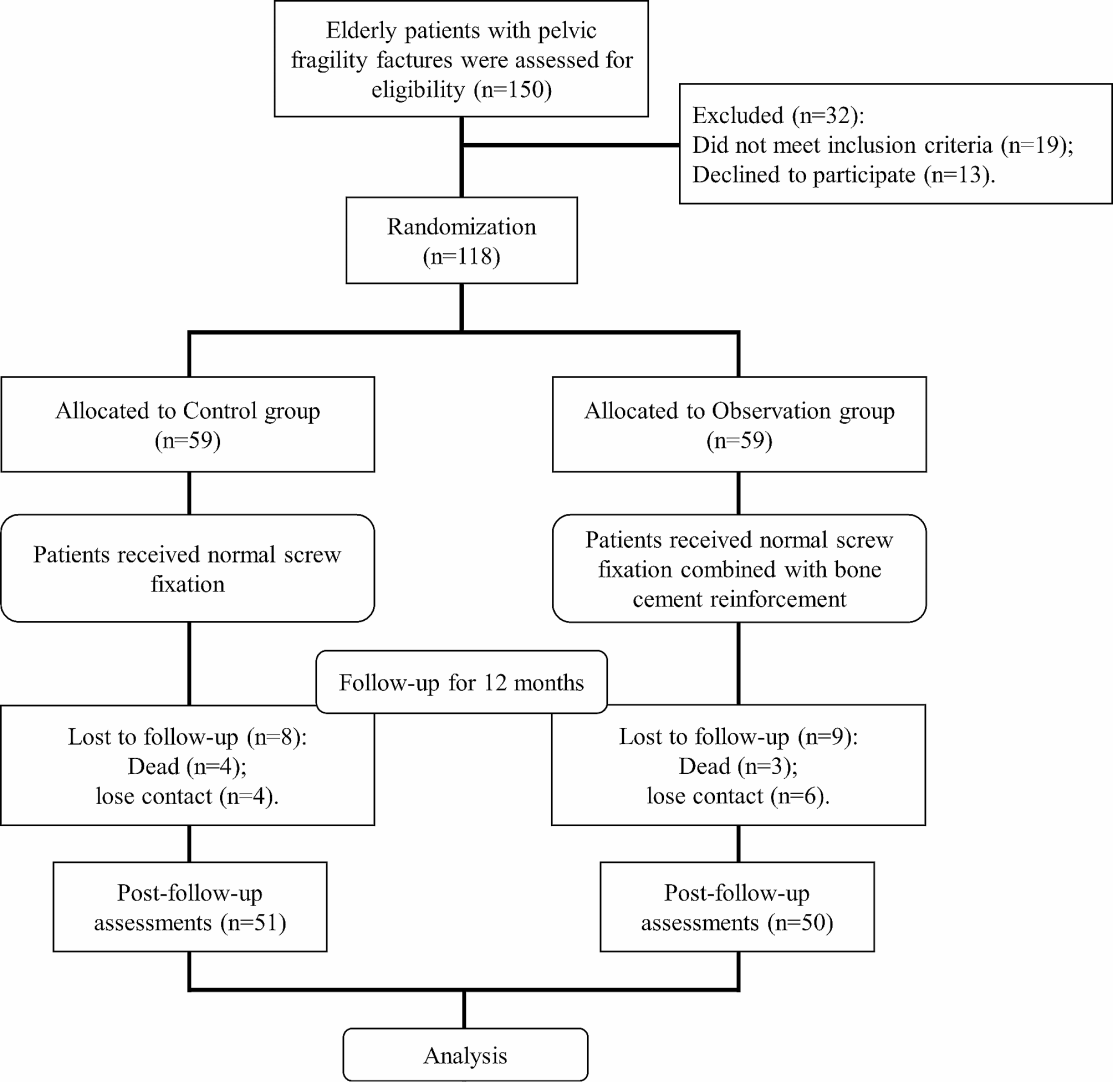
Research framework of this trail

In this study, a total of 150 elderly patients with pelvic fragility fractures were included in the evaluation, of which 19 were not eligible for inclusion, and 13 refused to participate. The 118 eligible patients were divided into two groups by random number table, namely the observation group (Observation) and the control group (Control). Artificial intelligence (Tianji Orthopedic Robot) was used to implant the anterior and posterior ring fracture cannulated screws for fixation. After the screw channel was established, the observation group was implanted with bone cement (#CLVP, Dezhou Jianjie Medical Equipment Co., LTD, Dezhou, China) using the spinal vertebral body forming sleeve system, and immediately implanted the hollow screw to complete the bone cement reinforcement. The control group underwent conventional treatment, which involved the implantation of cannulated screws to stabilize the fractured end of the pelvis. All patients were followed up for 1 year after treatment, during which 4 patients died and 4 lost contacts in the control group; 3 patients died and 6 lost contacts in the experimental group. Finally, 50 cases in the Observation group and 51 cases in the Control group was collected with complete follow-up data (Fig. 1). All participants have signed informed consent, and the study was approved by Cangzhou Hospital of Integrated Traditional Chinese Medicine and Western Medicine. The study was performed in strict accordance with the Declaration of Helsinki, Ethical Principles for Medical Research Involving Human Subjects.

Inclusion Criteria:

1. Participants aged 60 years or older.
2. Pelvic fractures resulting from low-energy injuries, such as falls from a standing position.

Exclusion Criteria:

1. Patients younger than 60 years with high-energy trauma.
2. Pelvic fractures caused by pathological conditions, including metastatic tumors, hormone-related fractures, and open fractures.
3. Participants with incomplete clinical or follow-up data.
4. Fractures located in other regions of the lower limbs.

### Surgical procedure

Observation Group: After general anesthesia, the patients were placed in a supine position, and standard sterilization and draping were performed. For patients with significantly displaced pelvic fragility fractures, bone traction reduction was employed. The surgical procedure included the following steps: (1) 3D Image Acquisition: 3D images of the pelvic region were obtained and transferred to the Tianji screen. (2) Planning and Screw Placement: Suitable entry points and directions for screw insertion were selected to complete the planning of the target screws. (3) Tianji Orthopedic Robotic Arm Execution: The Tianji robotic arm was used to perform screw guide K-pins insertion to verify the position first. The spine vertebral body shaping cannula system was used to implant bone cement, followed by the insertion of the hollow cannulated screws under the guidance of fine guide pins, completing the technique of cement reinforcement at the screw end.

Control Group: The Tianji robotic system was used for image acquisition, planning, and conventional implantation of hollow cannulated screws for fixation.

### Fracture healing assessment

Fracture healing was evaluated based on follow-up radiological findings and physical examinations. X-rays revealed a blurred fracture line with evidence of continuous callus formation. Additionally, there were no tenderness or axial percussion pain detected during the physical examination at the affected site, leading to the assessment of clinical healing of the fracture. It should be noted that our primary comparison focuses on clinical healing time and does not include functional aspects such as Majeed functional scores. The determination of patients’ clinical healing time in our study primarily relied on imaging data reviewed by specialized orthopedic and radiology professionals.

### Majeed score

The Majeed Pelvic Score, a specific scoring system introduced in 1989, encompasses five aspects: pain, work, sitting, sexual activity, and standing, which is further divided into walking aids, unassisted gait, and walking distance. Each aspect is graded from excellent to poor, with higher scores indicating better patient recovery. The criteria for the Majeed Pelvic Score are as follows: for pre-injury employed individuals, a perfect score is 100 points, with scores > 85 considered excellent, > 69 to 85 good, > 55 to 69 fair, and ≤ 55 poor; for pre-injury unemployed individuals, the maximum score is 80 points, with scores > 70 considered excellent, > 55 to 70 good, > 45 to 55 fair, and ≤ 45 poor. According to the “Guidelines for Minimally Invasive Surgery for Pelvic Fractures in China (2021)”, the Majeed score is a commonly used method for assessing postoperative efficacy and follow-up of pelvic fractures.

### Visual analog scale (VAS)

VAS is a utilized tool of pain assessment. At one end of the scale, patients are presented with a descriptor indicating “no pain,” while the opposite end represents the most intense pain imaginable. Patients are then instructed to place a mark on the line that corresponds to their current pain level, with the distance from the “no pain” end serving as a numeric representation of pain intensity. Typically, this distance is measured in millimeters or centimeters, allowing for precise pain quantification. A score of 0 indicates no pain, and a score of 10 indicates unbearable severe pain.

### Statistical analysis

SPSS 19.0 was utilized for data analysis. The data are presented as median (interquartile range) or n (percentage), and statistical comparisons were conducted using the Mann-Whitney test or Fisher’s exact test or Chi-square test.

## Results

### Demographic and clinical characteristics of the participants

Table 1 presents the demographic and clinical characteristics of patients with pelvic fragility fractures who underwent treatment with normal screw fixation (Control) or a combination of normal screw fixation and bone cement reinforcement (Observation). The analysis demonstrates that there were no statistically significant differences in age, BMI, course of disease, gender distribution, cause of injury, FFP classification, diabetes mellitus, and hypertension between patients treated with normal screw fixation and those who received combined treatment with bone cement reinforcement (See Table 1).

**Table 1:** Demographic and clinical characteristics of pelvic fragility factures patients received the treatment of normal screw fixation (Control) or combined with bone cement reinforcement (Observation)

Characteristics	Study group		p value
	Control (*n* = 51)	Observation (*n* = 50)	
Age (years)	69 (66, 76)	70.5 (65, 76.25)	0.806
Body mass index (kg/m^2^)	23.64 (21, 27.65)	23.07 (20.73, 26.15)	0.372
Course of disease (days)	10 (7, 13)	11.5 (7, 14.25)	0.676
Gender			
Male	19 (37.3%)	23 (46%)	0.423
Female	32 (62.7%)	27 (54%)	
Cause of injury			
Sitting fall	19 (37.3%)	22 (44%)	0.524
Standing fall	27 (52.9%)	21 (42%)	
Traffic accident	5 (9.8%)	7 (14%)	
FFP classification			
II	24 (47.1%)	21 (42%)	0.497
III	18 (35.3%)	23 (46%)	
IV	9 (17.6%)	6 (12%)	
Diabetes mellitus			
Yes	16 (31.4%)	13 (26%)	0.661
No	35 (68.6%)	37 (74%)	
Hypertension			
Yes	21 (41.2%)	24 (48%)	0.551
No	30 (58.8%)	26 (52%)	

The data are presented as n (percentage) or median (interquartile range). The comparisons of data were done by Mann-Whitney test or Fisher’s exact test or Chi-square test. FFP: Fragility fractures of the pelvis

### Healing time and time in bed of the participants

**Fig. 2:**
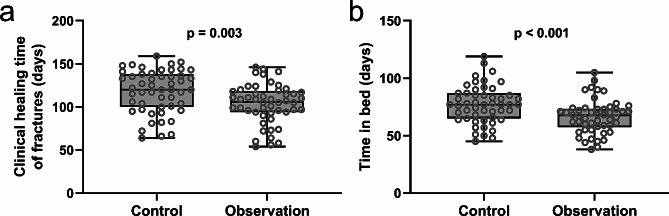
Comparisons of clinical healing time of fractures **(a)** and time in bed **(b)** of pelvic fragility factures patients received the treatment of normal screw fixation (Control, *n* = 51) or combined with bone cement reinforcement (Observation, *n* = 50). The data were shown with box plot. p values were calculated from Unpaired t test with Welch’s correction

The study compared the clinical healing time of fractures and the duration of bed rest between patients with pelvic fragility fractures who received either standard screw fixation (Control, *n* = 51) or a combination of standard screw fixation and bone cement reinforcement (Observation, *n* = 50). As shown in Fig. 2a, the clinical healing time of fractures in the observation group was significantly lower than that in the control group (*p* = 0.003), suggesting that bone cement reinforcement screws assisted by Tianji Orthopedics Robot accelerated the healing of fractures in the elderly. Similarly, the bed rest time of patients in the observation group was also significantly lower than that in the control group (*p* < 0.001), suggesting that the bone cement reinforcement screw assisted by the Tianji Orthopedic Robot is more beneficial for patients’ postoperative recovery compared to traditional technology (Fig. 2b).

### Patient’s pain level post-surgery

**Fig. 3:**
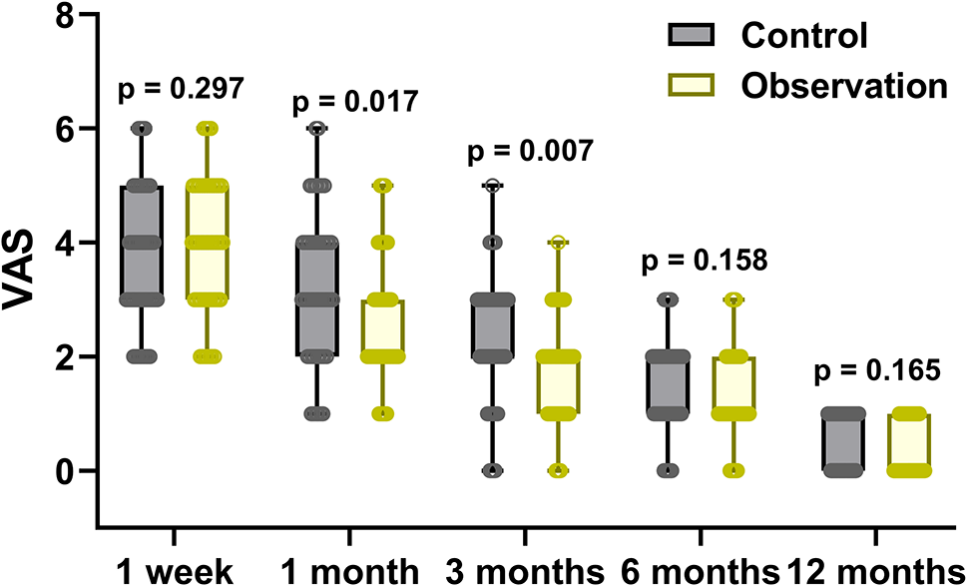
Comparisons of VAS at 1 week and 1, 3, 6, 12 months after surgery of pelvic fragility factures patients received the treatment of normal screw fixation (Control, *n* = 51) or combined with bone cement reinforcement (Observation, *n* = 50). The data were shown with box plot. p values were calculated from Mann Whitney test

The patients’ pain was assessed using the Visual Analog Scale (VAS) at 1 week, 1 month, 3 months, 6 months, and 12 months after surgical treatment (Fig. 3). The results revealed that there were no significant differences in pain between the two groups at 1 week after surgery. However, at 1 and 3 months after surgery, the Observation group experienced significantly less pain compared to the Control group. Notably, there was no significant difference in pain between the two groups at the 6-month postoperative evaluation. This analysis suggests that incorporating bone cement reinforcement in the Observation group resulted in reduced postoperative pain at 1 and 3 months, although the disparity in pain levels between the two groups lessened by the time of the 6-month follow-up.

### Functional recovery of the pelvis after fracture surgery

**Fig. 4:**
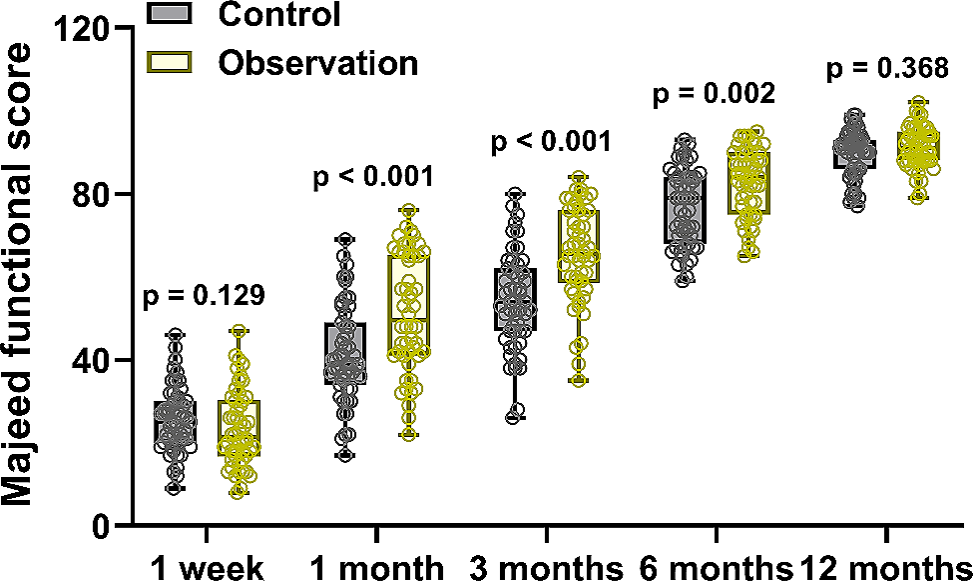
Comparisons of Majeed functional scores at 1 week and 1, 3, 6, 12 months after surgery of pelvic fragility factures patients received the treatment of normal screw fixation (Control, *n* = 51) or combined with bone cement reinforcement (Observation, *n* = 50). The data were shown with box plot. p values were calculated from Mann Whitney test

The patients’ postoperative functional outcomes were assessed using the Majeed functional scores at 1 week, 1 month, 3 months, 6 months, and 12 months after surgery. The results revealed that there were no significant differences in postoperative functional outcomes between the two groups at 1 week after surgery (Fig. 4). However, at 1, 3, and 6 months after surgery, the patients in the Observation group showed significantly better postoperative functional recovery compared to the Control group. Notably, by the 12-month postoperative evaluation, there were no significant differences in postoperative functional outcomes between the two groups. This analysis indicates that the use of bone cement reinforcement in the Observation group contributed to better postoperative functional recovery at 1, 3, and 6 months, but the difference in functional outcomes between the two groups diminished by the 12-month follow-up.

### Typical imaging of patients in the observation group and control group

**Fig. 5:**
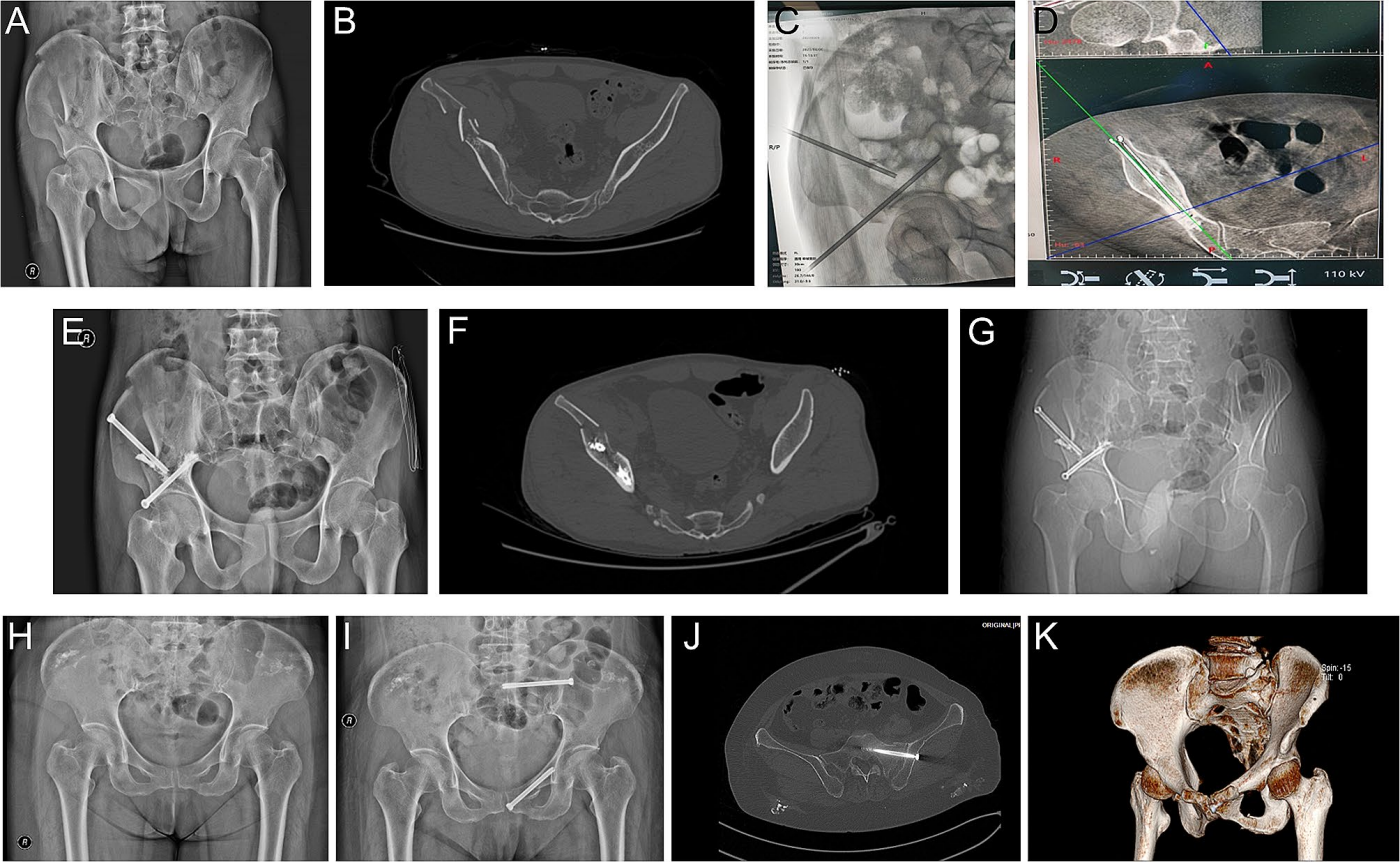
Typical cases from observation group **(A-G)** and control group **(H-K)**. A, Preoperative X-ray and CT films **(B)** of the pelvis from one case in observation group. **C**, Schematic diagram of the bone cement cannula implanted into the desired position. **D**, Schematic diagram of hollow nail implanted into the place of cement reinforcement. **E**, Post-operative X-ray and CT films **(F)** of the pelvis from that case in observation group. **G**, Post-operative holistic CT radiograph from that case in observation group. **H**, Preoperative X-ray film and post-operative X-ray film **(I)** from one case in control group. **J**, Post-operative CT plain scan of pelvis from that case in control group. **K**, Post-operative CT reconstruction of the pelvis from that case in control group

Figure 5 presents representative preoperative and postoperative imaging of patients from the Observation and Control groups. Figure 5a-g were from the Observation group, while Fig. 5h-k were from the Control group. The preoperative anteroposterior radiograph of the pelvis of a patient in the observation group (Fig. 5a) was shown first. Figure 5b was the result of plain CT scan of the pelvis before operation. During the operation, the bone cement sleeve was implanted at the predetermined position (Fig. 5c), and the cannulated screw was implanted to the bone cement reinforcement (Fig. 5d). The postoperative anteroposterior X-ray film of the pelvis (Fig. 5e) and the postoperative CT scan of the pelvis (Fig. 5f) showed the position of the cannulated screw. Figure 5g was postoperative CT image (holistic view). Figure 5h was the preoperative anteroposterior X-ray of the pelvis in the control group; Fig. 5i was the postoperative anteroposterior X-ray of the pelvis in the control group; Fig. 5j was the plain scan of the pelvis in the control group after CT (screw implantation in the sacroiliac joint); Fig. 5k was the reconstruction of the pelvis in the control group after CT. Generally, we believe that patients who have undergone surgery assisted by bone cement reinforcement screws assisted by the Tianji Orthopedics Robot tend to have better surgical outcomes compared to those who have undergone traditional surgery.

## Discussion

The management of pelvic fragility fractures in elderly patients remains a complex challenge for orthopedic and geriatric medicine [18, 19]. As the elderly population continues to grow, the incidence of these fractures is expected to rise, making it crucial to identify effective treatment strategies to improve patient outcomes [20]. This study aimed to investigate the application effect of bone cement reinforcement technology assisted by artificial intelligence (Tianji Orthopedic Robot) in elderly patients with pelvic fragility fractures.

The role of artificial intelligence (AI) in bone surgery represents a significant advancement in orthopedic surgery, particularly in the treatment of complex fractures and deformities [21, 22]. AI technologies, such as advanced imaging analysis and computer-assisted planning tools, play a crucial role in the preoperative phase. AI algorithms can process and analyze complex medical images, such as CT scans or MRI data, with greater speed and accuracy than human capabilities [23, 24]. This allows for detailed three-dimensional reconstructions of the affected bone, providing surgeons with a comprehensive view of the fracture or deformity [25]. AI-powered planning tools can assist in optimizing the surgical strategy by simulating various fracture scenarios and predicting potential outcomes [26]. These predictive capabilities help surgeons choose the most suitable surgical parameters, such as the rate and rhythm of bone cement reinforcement to achieve the desired surgical goals while minimizing potential complications [27]. During the surgery itself, AI-driven robotic systems have emerged as powerful tools to enhance precision and control [28]. AI-powered robotic arms can execute the planned bone cement reinforcement process with unparalleled accuracy, minimizing the risk of error and maximizing the efficiency of the surgery [29]. By integrating real-time feedback mechanisms, AI-enabled robotic systems can continuously monitor the bone cement reinforcement process, making necessary adjustments based on intraoperative conditions [30]. This closed-loop feedback mechanism ensures optimal bone alignment and minimizes soft tissue damage, contributing to improved surgical outcomes [31].

The results of this controlled clinical trial demonstrated that the bone cement reinforcement technique, combined with the use of the Tianji Orthopedic Robot, offered significant advantages in the management of pelvic fragility fractures in the elderly. The findings revealed that the patients in the Observation group, who received bone cement reinforcement with the robotic assistance, experienced accelerated clinical healing time of fractures compared to those in the Control group. Additionally, the Observation group showed reduced time spent in bed after surgery, suggesting enhanced postoperative recovery. These outcomes emphasize the potential of bone cement reinforcement to promote efficient fracture healing and early mobilization in elderly patients, thereby minimizing the burden of bed rest and improving overall functional outcomes.

Furthermore, the evaluation of patients’ pain levels using the VAS provided valuable insights into the postoperative pain experience. The findings demonstrated that the patients in the Observation group reported significantly less pain at 1 and 3 months after surgery compared to the Control group. This indicates that the bone cement reinforcement technique contributed to improved pain relief during the early postoperative period, which is crucial for patient comfort and mobility. However, it is important to note that by the 6-month follow-up, there was no significant difference in pain between the two groups. This suggests that the initial pain reduction observed in the Observation group might have subsided over time, indicating the need for further investigation into the long-term pain outcomes.

Moreover, the assessment of postoperative functional outcomes using the Majeed Score revealed that patients in the Observation group demonstrated superior functional recovery at 1, 3, and 6 months after surgery compared to the Control group. The bone cement reinforcement technique, combined with robotic assistance, appeared to facilitate better functional outcomes during the early postoperative period. However, interestingly, by the 12-month follow-up, there were no significant differences in functional outcomes between the two groups. This may indicate that the initial benefits observed in the Observation group reached a plateau over time, warranting further investigation into the long-term functional recovery and durability of the bone cement reinforcement technique.

The typical imaging presented in Fig. 5 provided a visual representation of the surgical procedure and the postoperative outcomes in both groups. The images showcased the successful implementation of bone cement reinforcement with the robotic assistance in the Observation group, highlighting the precision and efficacy of this technique in stabilizing pelvic fractures. The comparison of imaging between the Observation and Control groups underscored the advantages of bone cement reinforcement in promoting fracture healing and achieving satisfactory postoperative outcomes.

While this study provided valuable insights into the application of bone cement reinforcement with robotic assistance in elderly patients with pelvic fragility fractures, several limitations warrant consideration. First, the sample size was relatively small, which might affect the generalizability of the findings. A larger multicenter study would be beneficial to validate the results and draw more robust conclusions. Additionally, the follow-up duration was limited to 12 months, and longer-term outcomes beyond this period were not evaluated. Considering that pelvic fragility fractures can have prolonged implications for elderly patients, extended follow-up assessments are necessary to assess the durability of the bone cement reinforcement technique and its impact on functional recovery and pain relief.

## Conclusions

In conclusion, the results of this study demonstrate that bone cement reinforcement with the assistance of the Tianji Orthopedic Robot offers significant advantages in the treatment of elderly patients with pelvic fragility fractures. The technique was associated with accelerated clinical healing time, reduced time spent in bed after surgery, and improved postoperative pain relief and functional recovery during the early postoperative period. However, the initial benefits observed in the Observation group may have diminished over time. Further studies with larger sample sizes and longer follow-up durations are required to establish the long-term effectiveness and durability of the bone cement reinforcement technique in this specific patient population. The findings from this study contribute to the ongoing research and advancement of treatment options for pelvic fragility fractures in the elderly, aiming to enhance patient outcomes and improve their quality of life.

## Data Availability

The raw data supporting the conclusions of this article will be made available by the authors, without undue reservation.
